# Evaluating the acceptability, feasibility, and appropriateness of treatment engagement strategies for primary care patients at risk for suicide: a pilot study

**DOI:** 10.3389/fpsyt.2026.1897475

**Published:** 2026-08-05

**Authors:** Courtney Benjamin Wolk, Biiftu Duresso, Samantha E. Weiss, Gabriela Kattan Khazanov, Molly Candon, David W. Oslin, Esha Patel, Matthew J. Press, Alison Buttenheim, Eleanor Anderson, Matteo Pieri, Shari Jager-Hyman

**Affiliations:** 1 Department of Psychiatry, University of Pennsylvania Perelman School of Medicine, Philadelphia, PA, United States; 2 Leonard Davis Institute of Health Economics, University of Pennsylvania, Philadelphia, PA, United States; 3 Corporal Michael J Crescenz Department of Veterans Affairs Medical Center, Philadelphia, PA, United States; 4 Department of Medicine, University of Pennsylvania Perelman School of Medicine, Philadelphia, PA, United States; 5 Department of Family and Community Health, University of Pennsylvania School of Nursing, Philadelphia, PA, United States

**Keywords:** collaborative care, engagement, implementation science, mental health, primary care, suicide

## Abstract

**Introduction:**

Many patients at risk for suicide do not initiate mental health treatment following referral by a primary care provider. The Collaborative Care Model, an evidence-based approach to integrating mental health services within primary care, is a promising approach for identifying and managing patients at risk for suicide. This study sought to develop strategies for increasing Collaborative Care treatment engagement in partnership with key interest-holders.

**Methods:**

In this multi-arm non-randomized feasibility pilot trial, we used a behavioral mapping and rapid prototyping process to develop and then pilot four strategies for engaging primary care patients at risk for suicide in Collaborative Care: Caring Contacts, reminders, an informational poster, and motivational interviewing. Strategies were rolled out sequentially via routine care and their acceptability, feasibility, and appropriateness were rated by patients using validated measures.

**Results:**

Primary care patients (n = 29) with documented risk for suicide who were exposed to a strategy completed research measures. Strategies were rated as moderately to highly acceptable, with reminders and Caring Contacts being rated most acceptable, feasible, and appropriate. Motivational interviewing was rated least acceptable, feasible, and appropriate, and few patients interacted with the informational poster.

**Discussion:**

Results suggest personalized electronic communications, such as reminders and Caring Contacts messages, may be perceived more favorably by patients at risk for suicide. Future research that tests these promising strategies in a fully powered trial design is needed.

**Trial registration:**

https://clinicaltrials.gov/, identifier NCT05021224.

## 1 Introduction

Despite increased awareness and suicide prevention efforts, suicide rates remain high, especially for historically marginalized groups (1). Studies have found that most people who die by suicide receive primary care services in the year prior to death (2), highlighting the importance of primary care as an access point for suicide prevention services. Primary care has additional advantages over specialty mental health settings, including greater accessibility, reduced stigma, and the opportunity to leverage established provider-patient relationships.

The Collaborative Care Model (CoCM) is one widely implemented, evidence-based approach for integrating mental health services within primary care. CoCM increases access to mental health services and has been associated with reductions in suicidal ideation (3, 4). However, many patients at risk for suicide do not initiate mental health treatment following referral by a primary care provider. Emerging evidence suggests that patients reporting more frequent suicidal ideation are less likely to engage in mental health care than those with less frequent or no suicidal ideation (5, 6), possibly due to lower levels of functioning and greater depression severity and/or hopelessness, emphasizing the need for strategies that bridge the referral-to-treatment gap. Although strategies, such as reminders and motivational interventions, have shown promise in enhancing mental health treatment engagement overall (7–9), the strength of evidence varies by population, care setting, and implementation approach. More work is needed to address the diverse barriers to mental health treatment engagement among patients at risk for suicide specifically as this population has rarely been a focus in the treatment engagement literature.

Given the public health crisis of suicide, methods to accelerate the development and implementation of feasible, acceptable engagement strategies are needed. Rapid prototyping, a method for briefly testing and refining intervention and implementation strategies to quickly determine feasibility and acceptability, can reduce the time and cost usually required to develop and implement interventions in real-world contexts (10, 11). The current study was part of a larger mixed-methods study aimed at increasing mental health treatment engagement among primary care patients at risk for suicide (12). As part of the parent study, we previously employed mixed methods (i.e., qualitative interviews, review of electronic health record data) to identify characteristics of patients at risk for suicide who did or did not engage in mental health treatment after referral, as well as barriers and facilitators to engagement (5, 6).

In the current study, we leveraged findings from this preliminary work to develop engagement strategies aligned with the EAST behavioral science framework, which posits that behavior change is most likely when the target behavior (in this case, treatment engagement) is Easy, Attractive, Social, and Timely (13). We then collaborated with a team of experts in implementation science, behavioral economics, suicide prevention, and primary and mental health care, to iteratively refine and pilot these strategies in primary care. The aims of the present study were to: (1) systematically develop and operationalize preliminary engagement strategies to increase initiation of mental health treatment among primary care patients at risk for suicide; and (2) evaluate the feasibility, acceptability, and appropriateness of these strategies among patients with elevated suicide risk referred to CoCM services.

## 2 Methods

In this multi-arm, non-randomized feasibility pilot trial, we piloted four discrete strategies to promote primary care patients’ engagement in mental health services [see (12) for additional study details]. The study is registered on ClinicalTrials.gov (NCT05021224) and all procedures were approved by the Institutional Review Board (approved protocol 844601). Study activities were conducted in partnership with a large CoCM program in a major metropolitan area in the Mid-Atlantic region of the United States [see (14) for full program details]. As part of usual care within this CoCM program, primary care patients referred for mental health services complete a standardized assessment and intake process and are scheduled with a mental health clinician in their primary care practice or supported in connecting with mental health care in the community.

The current study proceeded in two aims. First, we systematically identified and operationalized promising strategies for increasing engagement in mental health services. Then, we piloted each strategy to understand patient use and identify usability issues that may require refinement or discontinuation.

### 2.1 Aim 1: strategy development

To inform our strategy development, we sought to better understand the factors that impact mental health treatment engagement among primary care patients at risk for suicide. First, we used electronic health records to identify characteristics of at-risk patients who did and did not engage in mental health services following referral from their primary care clinician (6). Then, we conducted 70 semi-structured qualitative interviews with primary care and health system leaders, primary care and mental health clinicians, and patients to understand barriers and facilitators to mental health service engagement. Findings from these activities are reported in detail elsewhere (5, 6).

Next, we used a behavioral journey mapping procedure (15) to guide our development of a menu of engagement strategies, drawing from our quantitative and qualitative data, review of the literature, and expert consensus. Over the course of two half-day retreats conducted over videoconference, an expert in user-centered design led a panel of experts in implementation science (n = 1), behavioral economics (n = 2), suicide prevention (n = 2), primary care (n = 2), and mental health services (n = 2) through behavioral mapping procedures. We presented patient prototypes based on key patient characteristics and experiences gleaned from our formative quantitative and qualitative work and literature review.

The panel worked together to identify likely cognitive, behavioral, and affective experiences of patients represented by each prototype and then mapped out a patient journey from referral to treatment initiation for each prototype based on our preliminary research, clinical experiences, and the literature. Through review and discussion of the patient journeys, we identified and prioritized unique (i.e., prototype-specific) and common barriers and facilitators to engagement that served as the foundation for strategy identification. We then identified and operationalized concrete strategies that could mitigate common barriers and partnered with collaborative care program leaders to prepare for pilot testing.

### 2.2 Aim 2: piloting strategies

#### 2.2.1 Participants

We identified eligible primary care patients via electronic health record (EHR) review between June 2023 and January 2024 from practices with CoCM. Inclusion criteria were elevated score (i.e., item score ≥ 1) on item 9 of the Patient Health Questionnaire-9 (PHQ-9; i.e., “Thoughts that you would be better off dead or of hurting yourself in some way”), age 18 years or older, and able to read and understand English. Patients with a documented diagnosis of dementia within the past two years or experiencing active psychosis or cognitive impairment that precluded their ability to provide informed consent and/or required emergency services were excluded, as were patients who had previously opted out of EHR-based research recruitment. Given that we piloted multiple strategies, patients who had already received a prior engagement strategy were excluded from completing research measures for subsequent strategies. The target sample for each strategy pilot was five participants.

#### 2.2.2 Procedures

Engagement strategies were piloted between June 2023 and January 2024. Strategies were IRB-approved for piloting as part of routine care, thus, consent to receive strategies was not required. Adjustments were made to some strategies based on initial feedback or learnings post-deployment. Study participants were recruited via the EHR patient portal or by phone and completed survey measures electronically following exposure to an engagement strategy. Waiver of written documentation of consent for completion of research measures was approved by the IRB. Surveys were programmed in REDCap, a secure, web-based electronic data capture application (16), and participants were compensated $25 for completing the surveys.

#### 2.2.3 Measures

Acceptability, feasibility, and appropriateness of each strategy were assessed using the Acceptability of Intervention Measure (AIM*)*, Feasibility of Intervention Measure *(*FIM*)*, and Intervention Appropriateness Measure (IAM*)*, respectively (17). The AIM, FIM, and IAM are each validated, 4-item questionnaires assessing participants’ perception of an intervention (or in this case, engagement strategy) via a 5-point Likert scale ranging from 1 = “completely disagree” to 5 = “completely agree.” Item stems were tailored for each engagement strategy (e.g., “The message meets my approval,” “The reminder seems fitting,” “These materials seem easy to use”). A mean score is computed for each measure across the 4 items, with higher scores indicating more favorable perceptions.

#### 2.2.4 Data analysis

We computed descriptive statistics for participant demographics and self-report measures and visually inspected differences on the AIM, FIM, and IAM across strategies. Due to the small sample sizes in some conditions and preliminary nature of this pilot study, we did not conduct inferential statistics. Analyses were completed in IBM SPSS Statistics version 28.0.

## 3 Results

### 3.1 Aim 1: strategy development

**Figure 1:**
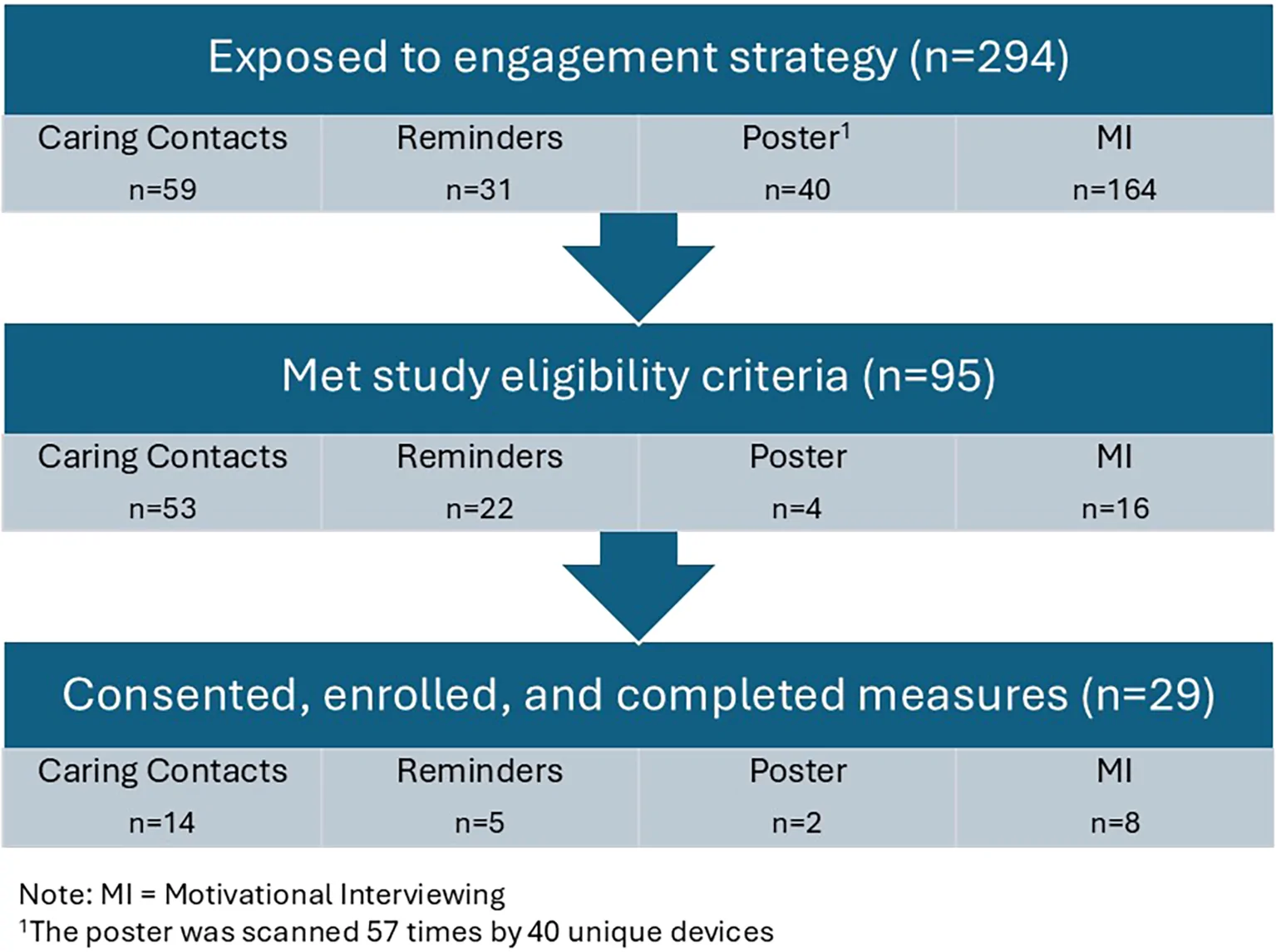
Consort diagram.

The following strategies were identified and operationalized for piloting. See the Supplemental File for examples of how each strategy was operationalized. See **Figure 1** for participant flow within each strategy. No patients contributed data to more than one strategy.

#### 3.1.1 Caring contacts

Caring Contacts are brief, non-demanding messages from the care team that have been shown to reduce suicidal ideation and attempts when sent to patients following discharge from acute care settings (18–20). On behalf of the treatment team, a bachelor’s-level clinical research coordinator sent Caring Contacts messages to 59 patients through the EHR communication system in June 2023. The study team then sent a separate message to 53 potentially eligible patients inviting them to participate in the study procedures. Of those invited, 16 consented and 14 completed the survey measures and were included in the analysis (see **Figure 1** for more information).

#### 3.1.2 Reminders

Appointment reminders aimed to help patients remember upcoming appointments and were sent 48 hours prior to a scheduled intake assessment or first appointment with a CoCM program provider. We piloted both neutral and gain-framed messages over the span of four weeks between June 2023 and July 2023, with initial messaging refined based on feedback from our clinician and leader partners. Both types of messages included the details of the upcoming appointments. The gain-framed messages contained additional language highlighting the benefits of attendance, based on previous research demonstrating that this approach increases appointment attendance for patients in specialty mental health care settings (21). Appointment reminders were sent to 31 primary care patients, 22 of whom were eligible and invited to participate in the study. Five patients consented, completed all survey measures, and were included in the analysis.

#### 3.1.3 Informational poster

To demystify the process of connecting to care, an informational poster inviting primary care patients to scan a QR code to learn more about the CoCM program was displayed in all exam rooms at one large family medicine practice over the span of 20 weeks between September 2023 and January 2024. The QR code directed patients to either an infographic or video providing information about the CoCM program and the importance of connecting to care. The infographic was deployed for 15 weeks and then replaced by the video, in an effort to explore if a video would increase engagement, which was available for five weeks. After viewing the infographic or video, all patients were invited to complete a brief contact form if they were interested in participating in the study. Because posters were publicly displayed, this engagement strategy was available to all patients, not just those at risk for suicide, and eligibility was determined following engagement. The QR code was scanned 57 times by 40 unique devices. A total of 25 participants completed the study interest form (18 who received the infographic and seven who received the video). Of these individuals, four met eligibility criteria and were invited to participate; two consented, completed the survey measures, and were included in the analysis.

#### 3.1.4 Motivational interviewing

Motivational Interviewing (MI) is an effective strategy for increasing patients’ motivation to change a target behavior (in this case, we aimed to increase motivation to engage in mental health care) (7, 22). The CoCM program’s intake and triage coordinators received a brief training and written companion guide on MI principles, with the guide refined following feedback from clinical leadership, to support patients in deciding to engage in mental health treatment following referral and developing a plan with specific steps to reach the target behavior of attending an appointment (23). The MI strategy was piloted during the last four weeks of our pilot trials from December 2023 to January 2024. Of the 164 patients who completed MI during this period, 16 were eligible and invited to participate in the study. Eight patients consented and completed study procedures.

### 3.2 Aim 2: strategy pilots

A total of 29 patients engaged with the pilot strategies and completed research measures (range = 2 to 14 patients per strategy). Study participants identified as female (n = 25; 86.2%), Black/African American (n = 16; 55.2%) or White (n = 11; 37.9%), and non-Hispanic (n = 26; 89.7%) and reported an average age of 43.5 (standard deviation (SD) = 14.5) years. Additional information on participants’ demographics by strategy can be found in **Table 1**.

**Table 1:** Participant demographics.

Characteristics	Full sample	Caring contacts	Reminders	Informational poster	Motivational interview
	*N* (%)	*n* (%)	*n* (%)	*n* (%)	*n* (%)
Participants (N)	29	14	5	2	8
Mean Age (SD)	43.5 (14.5)	40.7 (12.3)	49.6 (18.5)	54.0 (21.2)	41.8 (14.8)
Gender					
Female	25 (86.2)	13 (92.9)	5 (100)	1 (50.0)	6 (75.0)
Male	3 (10.3)	0 (0.0)	0 (0.0)	1 (50.0)	2 (25.0)
Other	1 (3.4)	1 (7.1)	0 (0.0)	0 (0.0)	0 (0.0)
Race					
White	11 (37.9)	5 (35.7)	1 (20.0)	0 (0.0)	5 (62.5)
Black/African-American	16 (55.2)	9 (64.3)	3 (60.0)	2 (100)	2 (25.0)
Other	2 (6.9)	0 (0.0)	1 (20.0)	0 (0.0)	1 (12.5)
Ethnicity					
Hispanic	2 (6.9)	2 (14.3)	0 (0.0)	0 (0.0)	0 (0.0)
Non-Hispanic	26 (89.7)	11 (78.6)	5 (100)	2 (100)	8 (100)
Unknown	1 (3.4)	1 (7.1)	0 (0.0)	0 (0.0)	0 (0.0)

**Figure 1** outlines the number of primary care patients referred to the CoCM program who were naturalistically exposed to each of the engagement strategies during usual care, those who evidenced elevated suicide risk and met study eligibility criteria, and those who consented and completed participation. Most notably, while the poster’s QR code was viewed 57 times by 40 unique users over a 20-week period, only 25 individuals completed the interest form, and only two met study eligibility criteria and completed research participation. As a result, the target enrollment of five participants was not reached due to a lack of eligible participants engaging with the poster and agreeing to participate.

#### 3.2.1 Acceptability, feasibility, and appropriateness of strategies

Overall, strategies were rated as moderately to highly acceptable (M = 3.9, SD = 0.8), appropriate (M = 3.8; SD = 1.0), and feasible (M = 3.7, SD = 1.0; **Table 2**) by patients on the AIM, IAM, and FIM, respectively. Among the four strategies, reminders were rated as most acceptable (M = 4.4, SD = 0.5), feasible (M = 4.3, SD = 0.7), and appropriate (M = 4.4, SD = 0.9; **Table 2**). Caring Contacts were rated as moderately to highly acceptable (M = 4.0, SD = 0.9), feasible (M = 3.8, SD = 0.9), and appropriate (M = 3.9, SD = 0.8). While ratings for the informational poster were also in the moderate to high range for acceptability (M = 3.9, SD = 0.2), feasibility (M = 4.0, SD = 0.0), and appropriateness (M = 4.0, SD = 0.0), only two eligible participants rated this strategy. Lastly, MI was rated least acceptable (M = 3.3, SD = 0.8), feasible (M = 3.2, SD = 1.3), and appropriate (M = 3.3, SD = 1.1).

**Table 2:** Descriptive statistics.

Variable	Total sample	Caring contacts	Reminders	Informational poster	Motivational interview
	*M (SD)*	*M (SD)*	*M (SD)*	*M (SD)*	*M (SD)*
Participants (N)	29	14	5	2	8
AIM (Acceptability)	3.9 (0.8)	4.0 (0.9)	4.4 (0.5)	3.9 (0.2)	3.3 (0.8)
FIM (Feasibility)	3.7 (1.0)	3.8 (0.9)	4.3 (0.7)	4.0 (0.0)	3.2 (1.3)
IAM (Appropriateness)	3.8 (1.0)	3.9 (0.8)	4.4 (0.9)	4.0 (0.0)	3.3 (1.1)

Items are scored on a 1–5 Likert scale where higher scores reflect more positive perceptions of acceptability, feasibility, and appropriateness on the Acceptability of Intervention Measure (AIM), Feasibility of Intervention Measure (FIM) and Intervention Appropriateness Measure (IAM), respectively.

## 4 Discussion

In this study, a panel of experts followed a behavioral mapping process to develop and refine four promising, scalable strategies to support primary care patients at risk for suicide in engaging in CoCM services. Building on prior community-partnered qualitative and quantitative work (4–6), we refined and pilot tested these engagement strategies with patients referred to integrated mental health services in primary care. The two strategies with highest acceptability ratings—appointment reminders and Caring Contacts—involve direct, individualized communication with patients. While reminders have been widely studied as a strategy to increase treatment initiation across many service types, this study is novel in examining reminders as a tool to promote mental health treatment initiation for primary care patients at risk for suicide. Similarly, while Caring Contacts have been associated with reduced suicide deaths and attempts following psychiatric inpatient discharge (24), this study extends Caring Contacts to the primary care setting as a strategy to promote mental health treatment engagement among those at risk for suicide.

Among patients who received each engagement strategy, the greatest proportion of those who received Caring Contacts messages from the primary care team agreed to participate in the study and complete research measures. While we cannot be sure that this was because Caring Contacts specifically promoted engagement in health services, this suggests Caring Contacts may be a promising avenue for future research. Although Caring Contacts were rated by patients as highly acceptable, feasibility ratings were slightly lower. Given the deliberate design of Caring Contacts to convey personalization rather than automation, they may have been perceived as resource-intensive. However, Caring Contacts messages can be deployed efficiently via the EHR with minimal resources. Future research should examine their implementation and effectiveness in promoting behavioral health treatment engagement for primary care patients.

Some strategies, like informational posters, had less engagement. Despite being placed in busy exam rooms for an extended time frame, few eligible patients scanned the QR code to access information about connecting to mental health care. One possibility is that this reflects limited utility of passive methods, like posters or flyers, compared to targeted direct messaging such as electronic reminders and Caring Contacts messages, for engaging patients at risk for suicide. However, this study was not powered to compare strategies head-to-head, and it is possible that observed differences were due to differing recruitment or delivery mechanisms. While MI was rated less favorably by patients, it may be that this strategy was harder for patients to isolate and rate, given it was incorporated into standard intake practices. It is also important to note that we were unable to monitor fidelity of MI implementation, thus we cannot be certain that participants received MI as intended.

This pilot study has several limitations to consider. We were unable to systematically assess the effectiveness of each strategy on first mental health visit attendance for two key reasons. First, engagement in specialty mental health services outside of the health system is not systematically documented in the EHR. Second, asking patients to report post-strategy treatment engagement would introduce potential confounding effects (e.g., a pending follow-up study visit might prompt a patient to initiate treatment) or result in a biased sample (e.g., patients who initiate treatment may also be more likely to attend a follow-up study visit). Additionally, given that this was a pilot trial, our small sample size limited our ability to comparatively evaluate the differences in acceptability, feasibility, and appropriateness between strategy groups. A randomized study design with a larger sample size to ensure sufficient power would be needed to evaluate the effectiveness of the strategies identified in this pilot study. Additionally, patient input was obtained prior to strategy development and patient feedback elicited after engagement with the strategies. However, patient participation in research is not equivalent to patient involvement in the research process, where people with lived experience are actively involved as partners throughout the study. Patients were not involved in the design, piloting process, analysis, results interpretation, or in developing study implications directly; doing so could have identified further opportunities for strategy refinement. Our team is actively engaging patient partners in our ongoing efforts to refine promising strategies for future testing, as we recognize the critical importance of incorporating perspectives of people with lived experience in this research. Finally, this study took place in a single health system; future work to explore generalizability in other health systems is needed.

This study is novel in its use of behavioral science and implementation science methods to develop strategies to support engagement of primary care patients at risk for suicide in CoCM services following referral from primary care. While preliminary, our findings signal that patients may respond better to direct messaging interventions compared to strategies deployed widely to all patients, such as posters in exam rooms. This is encouraging given the relative feasibility of deploying these types of direct messaging strategies widely using automation through the EHR. Future studies that test these promising strategies at scale and with adequate power are needed.

## Data Availability

The datasets presented in this study can be found in online repositories. The names of the repository/repositories and accession number(s) can be found below: https://nda.nih.gov/, collection doi: 10.15154/c2t3-xz18.
