# Supplementary material for: Evaluating the acceptability, feasibility, and appropriateness of treatment engagement strategies for primary care patients at risk for suicide: a pilot study
Source: Front Psychiatry. 2026 Aug 5;17:1897475. doi: 10.3389/fpsyt.2026.1897475 (PMC13488163; doi:10.3389/fpsyt.2026.1897475)
Supplement: Supplementary file 1 [file DataSheet1.docx]

**Supplemental File: Sample Strategy Materials**

1. Caring Contacts Message
2. Reminders
3. Informational Poster
   a. Infographic
   b. Informational video
4. Motivational Interviewing Guide
1. **Caring Contacts Message**

2. **Reminders**

**Intake Reminders**

Generic Message

“REMINDER: A Penn Medicine Integrated Care Intake Coordinator will reach out to you within the next few days. If you miss their call, please call them back to complete your intake assessment at 215-615-4222.”

Gain-Framed Message

“Penn Medicine Assessment Reminder: A Penn Medicine Integrated Care Intake Coordinator will reach out to you within the next few days. If you miss their call, please call them back to complete your intake assessment at 215-615-4222.

It is important to complete your assessment. If you complete the assessment, the Intake Coordinator will identify mental health services or resources that may be right for you.”

**Appointment Reminder**

Generic Message

“REMINDER: Penn Medicine appointment with [Provider] on [Date, Time] at [Location]. Questions? Contact 215-615-4222.”

Gain-Framed Message

“Penn Medicine Appointment Reminder: Appointment with [Provider] on [Date, Time] at [Location].

It is important to attend your appointment. If you decide to attend, your provider will work with you to help you get the most out of your mental health treatment and improve your well-being.

Questions? Contact 215-615-4222.”

**3. Informational Poster**

**3a. Infographic**

**3b. Informational video (screenshots)**

3. **Motivational Interviewing Guide**
